# Therapeutic Relational Communication and Resilience among Nursing Professionals in a Pandemic Situation

**DOI:** 10.3390/nursrep14030159

**Published:** 2024-08-26

**Authors:** Isabel Mª Sáez-Ruiz, Verónica V. Márquez-Hernández, Genoveva Granados-Gámez, Anabel Corral-Granados, Consuelo Artero-López, Lorena Gutiérrez-Puertas

**Affiliations:** 1 Hospital Universitario Torrecárdenas, 04009 Almería, Spain; isabelm.saez.sspa@juntadeandalucia.es; 2 Department of Nursing, Physiotherapy and Medicine, Faculty of Health Sciences, Universidad de Almería, 04120 Almería, Spain; genoveva@ual.es (G.G.-G.); lgp524@ual.es (L.G.-P.); 3 Research Group for Health Sciences CTS-451, Health Research Center, 04120 Almería, Spain; 4 Department of Education, Universidad de Almería, 04120 Almería, Spain; acg287@ual.es; 5 Hospital Universitario Poniente, 04700 Almería, Spain; consuelo.artero.sspa@juntadeandalucia.es; 6 Experimental and Applied Neuropsychology Research Group HUM-061, Health Research Center, 04120 Almería, Spain

**Keywords:** communication, COVID-19, care, nursing, resilience

## Abstract

Therapeutic relational communication has become a fundamental human resource in the nursing profession. The positive relationship between nurse-patient communication and resilience has been shown in several studies. However, these aspects can be affected in adverse circumstances. The purpose of this study was to identify the relationship between nurse-patient therapeutic relational communication and the resilience of nursing professionals in adverse circumstances. A cross-sectional study with a sample of 201 nursing professionals was conducted. This study found high levels of both therapeutic relational communication between nurses and patients as well as resilience among nurses. The sociodemographic variables of age and years of experience in the profession positively correlated with the data obtained through the measurement instruments. The co-relational analysis revealed a positive correlation between nurse-patient therapeutic relational communication and nurse resilience, and the regression analysis showed that levels of successful therapeutic communication skills were minimally explained by resilience. Nursing professionals who participated in this study demonstrated adequate levels of therapeutic relational communication with their patients as well as adequate levels of resilience required to cope with the challenges of the COVID-19 pandemic in their daily nursing practice. This study was not registered.

## 1. Introduction

Nursing practice is characterised by the mastery of communication skills that allow these professionals to provide care based on transmitting information clearly and concretely, inspiring confidence and proximity, respecting vital space, synchronising verbal and non-verbal language, listening actively, and showing authenticity [1]. This reality makes the nursing profession a reference in healthcare, being immersed in a dynamic care process that addresses the psychological, social and physical demands of the patient [2].

The experience of the COVID-19 pandemic since March 2020 [3,4,5] has led nursing professionals to deliver part of their care through what is known as digital health [6]. Consequently, in this situation, there has been a decrease in the quality of nurse-patient communication as part of the physical contact with the patient is lost during the care process [7,8]. This new reality favours the depersonalisation of care and causes the presence of communication barriers between the nurse and the patient [9]. Training in communication skills for relational supportive care shows direct benefits in the humanisation of care and the improvement of nursing professional performance [10,11]. Along these lines, relational communication skills have been used to explain the quality of nursing care [12]. Aspects related to the nursing professional, the context in which care is provided and the characteristics of the patient have been positively valued [13]. These elements explain the foundations of relational nurse-patient communication [14]. Specifically, among the characteristics of the professional, the capacity for nursing resilience has been considered a key element and a buffer against negative consequences in professional care [15,16,17].

Therapeutic relational communication is understood as the communication that the nursing professional establishes with the patient within a context of human, therapeutic, interpersonal and care relationships, and whose purpose is to understand and meet the patient’s needs [18]. Therapeutic relational communication is characterised as a tool in a process of continuous change, dynamic, complex, and related to the life context of the people involved in it [19]. This relational modality has become a specific skill and a fundamental human resource for the nursing profession. All nursing care carries with it a relational part, which emerges and is maintained through the professional’s communication with patients during the care process, through technical and/or therapeutic procedures. At the same time, it is involved in the human responses of the person in their health/illness cycle [12]. The relational quality of communication becomes evident when patients express the need and desire to be heard while receiving help [20].

Unfortunately, the modality of patient care during the pandemic crisis, together with internal and external workplace factors, has resulted in the appearance of communication barriers with negative effects on nurse-patient communication. Among the negative effects are nurses’ unwillingness to communicate with patients, the nurse’s negative attitude towards the patient, and the nurse’s lack of understanding of the patient’s psychological state [21]. Among the aggravating factors for the decrease in the quality of relational communication in crisis situations, the following has been found: increased prevalence of depression, post-traumatic stress, anxiety, low organisational commitment, job dissatisfaction, high burnout, and fatigue in nursing professionals [22,23,24,25].

Among the characteristics inherent to the professional that facilitate relational communication of help in crisis care situations is nursing resilience. This is considered a dynamic process that allows negotiating, managing, and adapting to pandemic, traumatic, or stressful situations, favouring better performance and work commitment [26,27,28]. Nursing resilience is a complex process that enables professionals to adapt to workplace stressors, avoid psychological harm and provide safe and high-quality care to patients [29,30]. The complexity of the process lies in maintaining balanced perceived social support, self-efficacy, optimism, humour, realism, and work-life balance [31]. In turn, an adequate and safe work environment supports the maintenance of resilience over time [17]. The global prevalence of nurse resilience is at low levels of 27% [24]. A study conducted on a sample of Spanish nurses found resilience levels of 45.2% [32]. Specifically, low resilience in Irish nursing was identified in association with weakened communication skills, high levels of emotional exhaustion, and depersonalised care [33]. Similar results were obtained in Chinese [27] and Mexican nursing [7]. Along these lines, the scientific evidence places value on the presence of resilient nurses in a continuously changing healthcare environment, such as the COVID-19 pandemic [16,17]. Based on the above, the lack of studies in the current healthcare context that relate the concept of therapeutic relational communication in nursing to the capacity for resilience justifies the purpose of the present study. The purpose of this research was to identify the relationship between nurse-patient therapeutic relational communication and the resilience of nursing professionals in adverse situations.

## 2. Materials and Methods

### 2.1. Design

A multiple linear regression study with a cross-sectional approach was conducted.

### 2.2. Setting and Participants

This study was carried out on a sample of nursing professionals from hospitals and public healthcare districts in Almeria (Andalucia, Spain). The nursing professionals who participated worked in hospitals in urban areas. These nurses worked in hospitalisation units with adult and paediatric patients, with 8-h shifts. The program Epidat 3.1 was used to calculate the sample size. For a population size of 400 nursing professionals, an expected proportion of 50%, a 95% confidence level, and an error of 5%, a sample size of 196 professionals was obtained. The final number of participants was 201.

The study followed a non-randomised convenience sampling system, with the following inclusion criteria: being a nursing professional, being employed as a nursing professional at the time of completing the questionnaire, and practising the profession in a hospital or primary care setting within Almeria. We excluded professionals with cognitive difficulties that would prevent them from completing the questionnaire.

### 2.3. Measurements

The instruments used were as follows:

The Nurse-Patient Therapeutic Communication Questionnaire (N-PTC questionnaire) [34]. This questionnaire analyses the communication of nursing professionals in nurse-patient therapeutic communication in the clinical setting, identifying the components involved in these interactions. It is composed of 49 items distributed into 3 dimensions. The questionnaire was developed following Peplau’s theoretical foundation. The main concepts involved in this theory are nursing, society or environment, health, and the human, and how they all interact, which is reflected in the dimensions of the developed questionnaire: (1) professional, (2) contextual and/or situational, and (3) patient. Specifically, the questionnaire allows assessment through the professional dimensions and patient elements collected in previous instruments such as respect or empathy. In contrast, this questionnaire takes into account the influence of other professionals on nurse-patient communication and interaction. In addition, it incorporates a situational/contextual dimension where environmental and cultural elements are represented. Each of the items was answered on a Likert scale from 1 (lowest value in agreement with the statement) to 4 (highest value in agreement with the statement). Items 9, 21, 24, 25, 35, 36, 39, 42, and 49 must be considered as inverse items. The higher the score, the greater the communication in each of the dimensions. The total score values were considered low (between 20 and 49 points), medium (between 50 and 98 points), medium-high (between 99 and 147 points), and high (between 148 and 196 points). Cronbach’s α coefficient for the original study was 0.90, and 0.92 in the present study.

The Connor-Davidson Resilience Scale [35] is a 10-item unidimensional scale (CD-RISC-10). This scale evaluates the capacity of individuals to thrive in the face of adversity. It is unidimensional and rated on a five-point Likert scale, which ranges from 0 (“Not true at all”) to 4 (“True nearly all the time”). The highest scores indicated the highest level of resilience. Cronbach’s α coefficient was 0.88.

The sociodemographic variables included were: sex, age, years in the profession, education, and the nursing unit where they were working during the pandemic.

### 2.4. Data Collection

The participants were informed of the voluntary nature of their participation in the study, as well as the confidentiality and anonymity of the data. The nursing supervisors of each work unit in the different health centres were contacted, and the questionnaires were sent to the nursing professionals both in paper format and digitally, using a web link and QR code. Participants signed an informed consent form prior to participation. The questionnaire was completed in 45 to 60 min. At the end, the participants were thanked for their collaboration in the study. The data collection process took place from January 2021 to January 2022.

### 2.5. Ethical Aspects

Permission was obtained from the Research Ethics Committee of Almería (16/2019). All participants signed an informed consent form and were informed of the voluntary nature of their participation. The Declaration of Helsinki and international guidelines on ethical aspects were followed.

### 2.6. Data Analysis

The SPSS version 27 statistical program was used for data analysis. First, a descriptive analysis of the sociodemographic variables of the sample was performed. The frequency and percentage of the categorical variables were calculated, while measures of central tendency and dispersion were obtained for the quantitative variables.

The Kolmogorov-Smirnov test was used to check whether the sample scores followed a normal distribution. Spearman’s correlation test was used to analyse the degree of correlation between quantitative variables. In addition, a multiple regression analysis was performed, considering a value of *p* < 0.05 as significant.

## 3. Results

### 3.1. Sociodemographic Characteristics of the Participants

The final sample consisted of 201 nursing professionals, of whom 81.1% (*n* = 163) were female and 18.9% (n = 38) male. The mean age of the participants was 40.35 years (SD = 12.41), and the mean number of years in the profession was 15.46 years (SD = 12.39).

Out of the total 201 professionals, 44.3% (n = 89) had a nursing certification, 30.8% (n = 62) held a Bachelor’s degree, 15.4% (n = 31) had a Master’s degree, 7.5% (n = 15) were specialists, and 2.0% (n = 4) were doctors. Regarding the participants’ nursing unit during the COVID-19 pandemic, 55.2% (n = 111) worked in a care unit free from COVID-19, 30.3% (n = 61) worked in a hospital unit with COVID-19 patients, and 14.4% (n = 29) in COVID-19 ICUs (Table 1).

### 3.2. Therapeutic Relational Communication

A total mean score of 151.02 (SD = 14.95) was found for the N-PTC questionnaire (Table 2), which showed a high score in therapeutic relational communication. A positive correlation was found between the N-PTC questionnaire and age (rs = 0.150; *p* = 0.034).

With regards to the dimensions of the questionnaire, the total mean score for dimension 1 (professional) was 64.73 (SD = 7.26); for dimension 2 (context), it was 44.04 (SD = 5.14); and for dimension 3 (patients), it was 42.24 (SD = 4.89) (Table 2).

### 3.3. Resilience

The total mean score on the CD-RISC-10 was 30.54 (SD = 5.42) (range 0–40), indicating a high level of resilience among the participating nurses. A moderate positive correlation was found when comparing the CD-RISC-10 scale with years in the profession (rs = 0.253; *p* = 0.000) and with age (rs = 0.219; *p* = 0.002).

### 3.4. Nurse-Patient Therapeutic Relational Communication and Nurse Resilience

A moderate positive correlation was found between nurse-patient therapeutic relational communication and nurse resilience (rs = 0.386; *p* = 0.000). There was also a moderate positive correlation between the different dimensions measured by the N-PTC questionnaire, which influence nurse-patient therapeutic relational communication and nurse resilience, the values being: dimension 1 (professional) rs = 0.368; *p* = 0.000, dimension 2 (context) rs = 0.314; *p* = 0.000, and dimension 3 (patient) rs = 0.305; *p* = 0.000 (Table 3).

After multiple linear regression analysis, it was observed that none of the sociodemographic variables were significant in predicting successful nurse-patient therapeutic relational communication. Resilience was significantly related to therapeutic relational communication (β = 1.158; *p* = 0.000). Finally, 17.6% of the variation in communication scores can be explained by the resilience variable.

## 4. Discussion

The aim of this study was to identify the relationship between nurse-patient therapeutic relational communication and the resilience of nursing professionals in a pandemic situation. In health crisis situations, the work context becomes compromised by the overwhelming number of patients and insufficient resources, which forces nurses to make an equal distribution of these resources, prioritising vital emergencies [36] In addition, nursing professionals experience thoughts of depression, anxiety, fatigue and job dissatisfaction when providing care in the midst of a health crisis [23,24,25].

The results obtained in this study indicate that the sample of nursing professionals studied had a high level of therapeutic relational communication skills. A similar result was obtained in a study conducted in Turkey, which determined high levels of communicative skills in nursing students [37]. However, other studies found average values for the level of communication of nursing professionals [38]. The findings of this study and similar studies show acceptable results in communication levels which may be the result of communication training programmes promoted by various research studies. Several studies continue to call for the need to implement training strategies in therapeutic relational communication [10,13].

When relating therapeutic relational communication skills with the variables studied, a positive correlation was observed between the N-PTC questionnaire and age. In line with the present study, Kim and Sim [36] observed in their research, an increase in the level of communication skills among nursing staff as age increased. However, other research found greater difficulty for younger nurses in establishing effective communication with their patients [39]. According to this study, this may be due to the existence of the organisations of authoritarian hierarchical systems and power differentials associated with seniority and professional status, in addition to young professionals with little experience. Hospital organisations should propose training programmes for young professionals that include patient communication management.

Adequate mean scores were obtained for the three dimensions of the N-PTC questionnaire (professional, context, and patient) in this study which can be considered high levels of therapeutic relational communication. The questionnaire compiles aspects involved in therapeutic relational communication, which were positively evaluated in recent research [13]. In addition, a work environment with an overwhelming nurse-patient ratio impacts nursing professionals, causing fatigue, poor performance, and job dissatisfaction, which decrease motivation to communicate effectively [40]. Also, managing the individual characteristics of patients allows nurses to satisfy and meet the communicative needs of these patients [13].

In the analysis of the N-PTC questionnaire by dimension, a positive correlation was found between the dimension that includes aspects of the nursing professional themselves, which may influence their communication skills, and age. In the research by Abdulla et al. [21], despite not finding a positive correlation between the age of the nursing professionals and their communication skills, a positive correlation was found between the personal aspect of lack of confidence and age. According to these authors, the majority of those aged 36–55 years agreed that nurses’ lack of self-confidence greatly affects communication, while participants aged 18–25 years and those aged 56 years and older gave a more mixed response. Finally, a positive correlation was found between certain communicative aspects of the nursing professional and years in the profession. In contrast to this result, Lee et al. [41] found no positive relationship between communication with the patient and years of practice in the profession.

Regarding the dimension that addresses aspects related to the work environment, two of the items obtained a medium-high mean score and measure aspects of interest to numerous researchers who have studied adverse circumstances. The first of these items, “*The context in which I carry out my activity impairs my communication with the patient*”, provides information that can be used to address the management of communication barriers due to the COVID-19 pandemic within a challenging work environment. One example of this is the use of masks, which impairs non-verbal communication by limiting facial expression and complicating the transmission of clear messages between the professional and the patient [42,43,44]. The second item was “*I am aware of communication difficulties that patients present in certain situations*”. These results may help professionals find solutions to communication barriers, such as those proposed in other studies, like taking into account environmental factors [45] or introducing the use of transparent masks [46], among others.

Finally, in the dimension that assesses aspects related to the patient that may influence communication skills, we find the items “*I notice the lack of training in therapeutic relational communication among colleagues in my clinical unit*” and “*The training in communication skills carried out in my unit has improved my relationship with patients*”. Both obtain a medium-high mean scores and provide information along the same lines as Lacerda et al. [47], who recognise the importance of training and mastering communication skills in daily practice to increase the quality and safety of care. Training strategies in therapeutic relational communication that encourage personal reflection, exchanging of opinions and open and respectful discussion should be promoted to stimulate personal development and. with it, the capacity for resilience. Incorporating this type of training could increase the quality of nursing care. Healthcare organisations and universities should be responsible for including these aspects in their training programmes.

Regarding resilience levels, the outcomes of this study showed high levels on the CD-RISC-10 scale. However, the results found by Gieniusz-Wojczyk et al. [48], showed that over half of nursing professionals obtained average levels of resilience, with only a small percentage reporting a high level. Moderate or mid-levels of resilience have been found in other studies [49,50]. The disparity in the level of resilience in different samples of nurses may be due to the fact that this healthcare group is often the most vulnerable to stress when compared to other healthcare workers [51]. Maintaining adequate levels of resilience in nurses makes it possible to mitigate stressful workplace issues, avoid psychological harm, and provide safe, quality care [25,31]. Healthcare organisations must support and promote the creation of courses aimed at professionals that provide the necessary tools to develop and maintain adequate levels of resilience. Just as procedural aspects require continuous training over time, aspects such as communication and resilience, among others, should be included in the training programmes of hospital centres.

With regard to the relationship between resilience and sociodemographic variables, a moderate positive correlation was found between resilience, age, and years in the profession. In several other studies, a correlation was found between resilience and age [49,50]. As for the variable of years in the profession, some studies show a positive correlation between this variable and resilience [48]. According to these studies, as age increases, so does exposure to workplace stressors, which may help develop psychological resilience. Overall, age appears to be an optimising factor for resilience.

Effective nurse-patient communication favours resilience among nursing professionals [51]. The present study showed a moderate positive correlation between relational communication skills and nurse resilience. Similarly, several studies have analysed the relationship between nurse-patient communication and resilience through different communication and resilience scales, the results of which support that communicative competence has a positive correlation with resilience [40,41]. Although all this research determined the existence of a positive correlation between communicative ability and resilience in nursing professionals, none of the specific studies addressed the therapeutic relational communication skills of nurses with patients.

### Limitations

The results of this research should be considered taking into account the following limitations. First, the sample selection was by convenience, so the results cannot be generalised. On the other hand, the adverse circumstances in which the data were collected made gaining access to the sample difficult. Finally, the scarce literature on therapeutic relationships and resilience made it difficult to discuss the results. As a future line of research, the measurement of the quality of nursing care could be incorporated into the analysis of therapeutic relational communication skills and nursing resilience.

## 5. Conclusions

The nursing professionals participating in this study demonstrated high levels of helpful relational communication skills with their patients and resilience in coping with the challenges presented by their daily practice. In this context, factors such as age and years in the profession were positively correlated with resilience and communication skills. A positive relationship was observed between both capacities, relational communicative ability to help and resilience, which is explained by the components of the dimension related to the professional.

The practical application of the results obtained in this study may favour the development of nursing training strategies in humanised care based on relational communication of help, as the mastery of this communicative skill favours personal reflection, the exchange of opinions, and open and respectful discussion to stimulate personal development, and thus the capacity for resilience. This training will, therefore, have a positive impact on the quality of nursing care, as nurses will be trained to provide care both in ideal working conditions and in times of health crisis.

## Figures and Tables

**Table 1:** Sociodemographic characteristics of the participants.

Variable	n	%
Sex		
Male	163	81.1
Female	38	18.9
Age	40.35 *	12.41 **
Years of Experience	15.46 *	12.39 **
Academic Training		
Diploma	89	44.3
Degree	62	30.8
Master’s Degree	31	15.4
Specialist	15	7.5
PhD	4	2.0
Unit of Work in Pandemic		
COVID-19 Free Unit	111	55.2
COVID-19 Hospitalisation	61	30.3
COVID-19 ICU Hospitalisation	2	14.4

* Mean ** Standard Deviation.

**Table 2:** Mean score and standard deviation of the N-PTC questionnaire and dimensions.

Dimension	M	SD	Range
Dimension 1. Professional	64.73	7.26	20–80
Dimension 2. Context	44.04	5.14	14–56
Dimension 3. Patient	42.24	4.89	15–60
Total N-PTC questionnaire	151.02	14.95	49–196

A positive correlation was evident when comparing dimension 1 with age (rs = 0.154; *p* = 0.029) and with years in the profession (rs = 0.168; *p* = 0.017).

**Table 3:** Correlation between the N-PTC questionnaire and CD-RISC-10.

		CD-RISC-10
Dimension	rs	*p*
Dimension 1. Professional	0.368 **	0.000
Dimension 2. Context	0.314 **	0.000
Dimension 3. Patient	0.305 **	0.000
Total N-PTC questionnaire	0.386 **	0.000

** *p* < 0.01.

## Data Availability

Dataset available on request from the authors.

## Public Involvement Statement

No public involvement in any aspect of this research.

## Guidelines and Standards Statement

This manuscript was drafted against the Strengthening the Reporting of Observational studies in Epidemiology for cross-sectional studies.
